# Let's get fISSical: fast *in silico* synchronization as a new tool for cell division cycle analysis

**DOI:** 10.1017/S0031182017000038

**Published:** 2017-02-07

**Authors:** BROOKE MORRISWOOD, MARKUS ENGSTLER

**Affiliations:** Department of Cell & Developmental Biology, University of Würzburg, Biocentre, Am Hubland, 97074 Würzburg, Germany

**Keywords:** cell division cycle, synchronization, automation, *Trypanosoma brucei*, organelles

## Abstract

Cell cycle progression is a question of fundamental biological interest. The coordinated duplication and segregation of all cellular structures and organelles is however an extremely complex process, and one which remains only partially understood even in the most intensively researched model organisms. Trypanosomes are in an unusual position in this respect – they are both outstanding model systems for fundamental questions in eukaryotic cell biology, and pathogens that are the causative agents of three of the neglected tropical diseases. As a failure to successfully complete cell division will be deleterious or lethal, analysis of the cell division cycle is of relevance both to basic biology and drug design efforts. Cell division cycle analysis is however experimentally challenging, as the analysis of phenotypes associated with it remains hypothesis-driven and therefore biased. Current methods of analysis are extremely labour-intensive, and cell synchronization remains difficult and unreliable. Consequently, there exists a need – both in basic and applied trypanosome biology – for a global, unbiased, standardized and high-throughput analysis of cell division cycle progression. In this review, the requirements – both practical and computational – for such a system are considered and compared with existing techniques for cell cycle analysis.

## INTRODUCTION

The cell division cycle is an area of fundamental biological interest, and the means by which individual cells duplicate and segregate their organelles underpins processes in both homoeostasis and disease. Trypanosomatids are outstanding model systems for the analysis of eukaryotic cell division cycle progression, due to their highly-polarized cellular structure, possession of several organelles in single-copy number only and the widespread availability of sophisticated molecular cell biology tools and reagents. They are in addition responsible for several neglected tropical diseases. Knowledge of the cell division cycle is thus important not only for understanding of their basic biology, but also for screening and characterizing both candidate and existing drugs. In this review, the current methods for cell division cycle analysis in the African trypanosome, *Trypanosoma brucei*, will be summarized and a new tool proposed for streamlining this process. The focus is on the replication of cellular organelles and structures rather than the signalling cascades regulating these processes, and the intention is to provide a wide-ranging piece that stimulates discussion and even debate within the broader research community.

## THE DISEASE IMPACT OF TRYPANOSOMA BRUCEI

Trypanosomatids are responsible for three of the 18 designated neglected tropical diseases (NTDs) (Hotez *et al.* 2014; World Health Organisation, 2016). Human African trypanosomiasis (HAT), Chagas disease and leishmaniasis respectively afflict members of the poorest human communities in sub-Saharan Africa, Central and South America, and the tropics. In addition, animal African trypanosomiasis depresses economic development and thereby acts to maintain a cycle of poverty (Shaw *et al*. 2014). HAT, colloquially known as sleeping sickness, is caused by two subspecies of *T. brucei* – *Trypanosoma brucei rhodesiense* (in East Africa) and *Trypanosoma brucei gambiense* (in West and Central Africa) (Franco *et al*. 2014). The latter is responsible for about 98% of all clinical cases and is currently the focus of an ongoing and highly successful World Health Organisation campaign; it is estimated that it can be eliminated as a public health concern by 2020 if current rates of decline continue (Simarro *et al*. 2015). While *T. brucei gambiense* HAT has traditionally been considered an anthroponotic disease, the existence of both animal reservoirs and asymptomatic human carriers is beginning to be debated (Sudarshi *et al.* 2014; Berthier *et al*. 2016; Welburn *et al.* 2016). *Trypanosoma brucei rhodesiense* HAT is a zoonosis and the parasite maintains a large reservoir in animals; it cannot therefore be eliminated, though the number of HAT cases it causes is much lower (Echodu *et al*. 2015). *Trypanosoma brucei* possesses an extremely sophisticated system of antigenic variation, which has consistently thwarted attempts to develop a vaccine; consequently, medical interventions have primarily relied on the use of pharmacological agents. The small number of available drugs and the complicated treatment regimens of existing ones make the need for new drugs an ongoing priority despite the encouraging news from affected areas (Drugs for Neglected Diseases Initiative, 2016).

## THE LIFE CYCLE AND MORPHOLOGY OF *T. BRUCEI*

*Trypanosoma brucei* is transmitted by its definitive host, the tsetse fly. Tsetse flies, which are haematophagous, become infected when feeding on trypanosome-infected mammals. Trypanosomes ingested in the blood meal will differentiate in the midgut lumen of the fly into the procyclic trypomastigote form (Vickerman, 1985; Sharma *et al*. 2009; Rotureau and Van Den Abbeele, 2013). The procyclic cells begin a migration through the tsetse fly, differentiating into mesocyclic forms along the way, until they ultimately reach the salivary glands. There, they differentiate into epimastigote forms and adhere to the salivary epithelium. Mammalian-infectious metacyclic forms are produced in the salivary glands and transmitted to potential mammalian hosts when the fly feeds. Once inside the mammalian tissues, the metacyclics differentiate into the slender bloodstream form (which, like the procyclic, has trypomastigote morphology) and begin to replicate. At high cell densities, a quorum-sensing mechanism stimulates slender bloodstream form cells to differentiate into the non-replicative stumpy form, which is optimized for transmission to the tsetse fly in order to complete the life cycle (Mony *et al*. 2014). Although *T. brucei* is primarily considered to inhabit the bloodstream, it is becoming apparent that populations in other tissues may play important roles in maintaining an infection and facilitating subsequent transmission. Its ability to cross the blood–brain barrier is well known, although the timing of this event may be sooner than previously thought (Frevert *et al*. 2012). Adipose tissue forms have also recently been described, which are metabolically distinct from bloodstream forms, colonize fat tissue, and may serve as reservoirs of infection (Trindade and Rijo-Ferreira *et al*. 2016). The presence of additional reservoirs in the skin has also been recently demonstrated (Caljon *et al.* 2016; Capewell and Cren-Travaillé *et al*. 2016). The existence of other morphological forms in tissues such as the skin is possible.

**Fig. 1.**
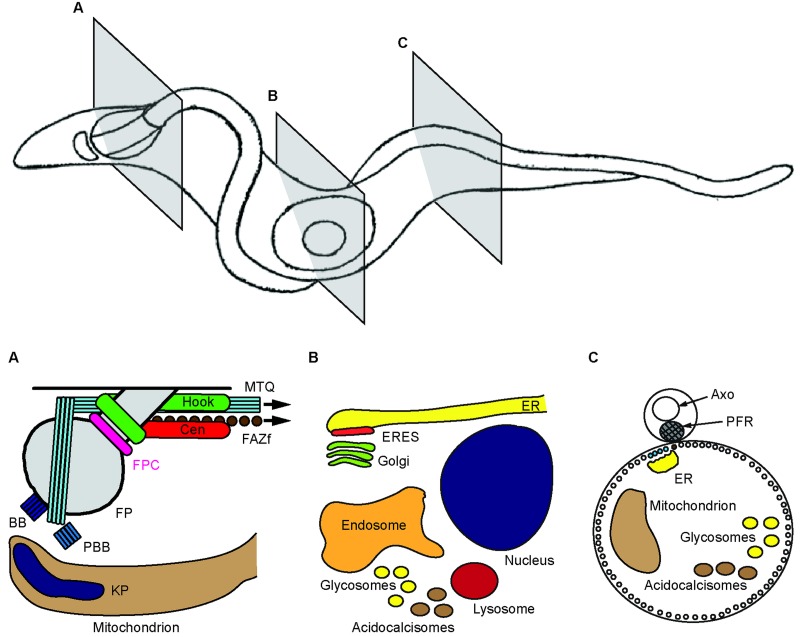
Cellular structures and organelles of *Trypanosoma brucei*. A drawing of a trypomastigote cell is shown, with three cross-sections (A–C) indicated. The drawing was made from a single frame of a video of a live, swimming, slender bloodstream form cell. (A) Schematic of structures and organelles in close proximity to the flagellar pocket (FP), shown in oblique cross-section. Indicated structures: basal body (BB), probasal body (PBB), flagellar pocket collar (FPC), hook complex (Hook), centrin arm (Cen), microtubule quartet (MTQ), flagellum attachment zone filament (FAZf). The mitochondrion and its genome, the kinetoplast (KP) are also shown. Note that the MTQ and FAZf extend beyond (arrows) the depicted cross-section, as does the mitochondrion. Not depicted: the tripartite attachment complex that links the BB to the KP. See text for details. (B) Schematic representation of cellular organelles, shown schematically in longitudinal cross-section. ER, endoplasmic reticulum; ERES, endoplasmic reticulum exit site. Note that the ERES and Golgi are in closer proximity to the flagellar pocket than depicted here. (C) Schematic of a transverse cross-section through an anterior part of the cell. The axoneme (Axo) and paraflagellar rod (PFR) within the flagellum are indicated. The ER and mitochondrion extend through the cell and are also shown here. Note that the ER is a network that extends throughout the cell; for simplicity, only the main branch associated with the flagellum attachment zone is depicted here. Note too that panels (A)–(C) are not shown at the same scale.

As noted above, procyclic, bloodstream and adipose tissue forms of *T. brucei* all share a trypomastigote morphology (Hoare and Wallace, 1966; Wheeler *et al*. 2013*a*). The cells are rod-shaped with tapered ends, with a single flagellum attached along the long axis and projecting slightly beyond the anterior tip of the cell (Fig. 1A–C). The cell shape, with its sharply-tapered posterior end and more gently tapering anterior part, is maintained by a dense corset of microtubules that lie underneath the plasma membrane (Gull, 1999). The root of the flagellum is within a small invagination of the plasma membrane found near the posterior end of the cell and termed the flagellar pocket (Lacomble *et al*. 2009) (Fig. 1A). Abutting the flagellar pocket membrane and nucleating the flagellar axoneme is a canonical eukaryote basal body, with an immature probasal body appended orthogonally to it (Lacomble *et al*. 2010). The flagellum contains an additional accessory structure, the paraflagellar rod, which runs parallel with and is tightly connected to the axoneme (Kohl *et al*. 1999) (Fig. 1C). Unlike the axoneme, the paraflagellar rod is not found within the portion of the flagellum within the flagellar pocket, and its posterior end is positioned at the point where the flagellum reaches the cell surface. Nucleated between the basal body and probasal body is a specialized quartet of four microtubules, which are thought to have the opposite orientation to the microtubules of the corset (Robinson *et al*. 1995). This microtubule quartet is wrapped diagonally around the flagellar pocket, and then extends underneath the flagellum to the anterior tip of the cell. Running alongside the microtubule quartet, and physically connected to the paraflagellar rod across the plasma membrane and flagellar membrane, is an electron-dense cytoskeletal filament. The microtubule quartet and the filament together comprise two elements of the flagellum attachment zone, which adheres the flagellum to the cell surface along a helical path (Vaughan *et al*. 2008; Sunter *et al*. 2015; Zhou *et al*. 2015). A recent review has proposed a five-domain descriptive model of the complete flagellum attachment zone (Sunter and Gull, 2016).

The plasma membrane can thus be divided into four morphologically distinct subdomains – the general plasma membrane, the flagellar membrane encasing the axoneme and paraflagellar rod, the flagellar pocket and the flagellar pocket neck (Lacomble *et al*. 2009). This last subdomain connects the flagellar pocket to the general plasma membrane, and is notable in that its membrane is tightly apposed to that of the flagellum. A number of cytoskeletal complexes are found in the flagellar pocket neck region (Fig. 1A). The best-characterized of these is an electron-dense cytoskeletal barrier element termed the flagellar pocket collar that is positioned at the boundary between the flagellar pocket membrane and the flagellar pocket neck membrane (Perdomo *et al*. 2015). Above the flagellar pocket collar are at least two other cytoskeletal complexes – the hook complex, which contains the repeat motif protein TbMORN1, and the centrin arm, which contains at least two of the five trypanosome centrin isoforms (Esson *et al*. 2012).

Within the cytoplasm, the cell contains the generic set of eukaryotic membrane-bound organelles, with some idiosyncrasies. The cell has a single mitochondrion, whose genome (the kinetoplast) is composed of an extremely topologically complex network of interlinked mini- and maxicircles of DNA (Fig. 1A). The kinetoplast is physically linked to the basal body by another transmembrane cytoskeletal complex (Ogbadoyi *et al*. 2003). The mitochondrion undergoes considerable morphological and metabolic reprogramming during the transition from the bloodstream form to the procyclic form of *T. brucei* (Vickerman, 1985; Zhang *et al*. 2010; Gunasekera *et al*. 2012). The single nucleus, with a diploid complement of DNA, is found anterior of the kinetoplast (Fig. 1B). The endoplasmic reticulum (ER) is dispersed throughout the whole cell volume, with a single ER exit site tightly apposed to the single-stacked Golgi complex on the posterior side of the nucleus and facing the flagellar pocket (He *et al*. 2004; Ho *et al*. 2006). All endo- and exocytic activity in *T. brucei* occurs on the flagellar pocket membrane (Grünfelder *et al*. 2003; Engstler *et al*. 2004). Internalized clathrin-coated vesicles are routed towards the endosomal–lysosomal network that is also present between the nucleus and the flagellar pocket (Engstler *et al*. 2004). Early endosomes are detectable as mostly circular cisternal structures. All cargo delivered to the early endosomes is eventually routed to the recycling endosomes, with a fraction passing first through the late endosomes. The recycling endosomes, which are structurally the most prominent of the endosomal organelles, are the principle recycling factories of the cell. The recycling endosomes use clathrin-coated vesicles to dispatch material to either the late endosomes or lysosomes; surface glycoproteins are recycled back to the plasma membrane in exocytic carriers. Vesicles with COPI or COPII coats mediate trafficking steps within the endomembrane system (Ho *et al*. 2006; Sevova and Bangs, 2009; Demmel *et al*. 2011). Other organelles include glycosomes, which contain the glycolytic enzymes, and acidocalcisomes, which are thought to be involved in osmoregulation (Opperdoes and Borst, 1977; Vercesi *et al*. 1994).

## CELL DIVISION CYCLE OF *T. BRUCEI*

**Fig. 2.**
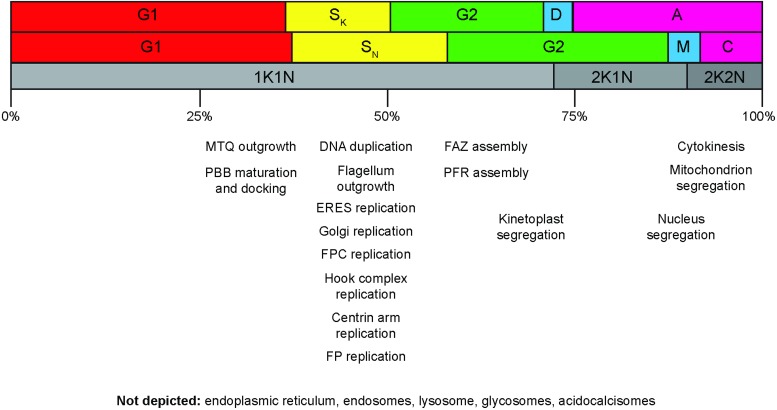
Cell division cycle progression and the replication of cellular structures and organelles. Top row: cell cycle phases for the kinetoplast. Canonical G1, S, G2 cell cycle phases are shown, along with the division period (D) and post-division period (A). Middle row: cell cycle phases for the nucleus. M, mitosis; C, cytokinesis period. Note that C includes both the post-mitotic phase and the actual cell cleavage period. Cell cleavage accounts for less than half of the total C period. The notation used in the top and middle rows is the standard 1990 terminology (Woodward and Gull, 1990). Bottom row: cell cycle classifications according to K/N counts. The approximate progression time through the whole cell cycle is indicated in percentages shown underneath. The approximate temporal position of cellular structure and organelle replication events are indicated. MTQ, microtubule quartet; PBB, probasal body; ERES, endoplasmic reticulum exit site; FPC, flagellar pocket collar; FP, flagellar pocket; FAZ, flagellum attachment zone; PFR, paraflagellar rod.

The cell division cycle of *T. brucei* has undergone extensive morphological characterization in procyclic and bloodstream form cells, which are the two most experimentally tractable stages of the life cycle (Sherwin and Gull, 1989; Wheeler *et al*. 2013*b*) (Fig. 2). The order of events is reckoned to be very similar, although the bloodstream form cells replicate faster (6 h in culture as opposed to 9 h for procyclics, approximately). The earliest documented event in the cell division cycle is the initiation of outgrowth of a new microtubule quartet. This is followed shortly by maturation of the probasal body, which docks with the flagellar pocket membrane and initiates outgrowth of a daughter flagellum (Lacomble *et al*. 2010). New probasal bodies are formed orthogonal to the old and new mature basal bodies. Replication – defined here as the process of duplication (doubling of mass) followed by segregation (separation into two distinct resolvable structures) – of cellular organelles such as the Golgi, ER exit site and endosomal–lysosomal system follows (Jeffries *et al.* 2001; Morgan *et al.*, 2001; He *et al*. 2004; Bangs, 2011). Duplication can be either templated or *de novo*. Templated duplication entails the old organelle or structure making a physical contribution to the new organelle or structure. *De novo* duplication utilizes only newly-synthesized material in which the organizational information is intrinsically coded. Replication of the flagellar pocket is coincident with an anticlockwise rotation of the new mature basal body around the pocket to leave it positioned posterior to the old basal body, flagellum and flagellar pocket (Lacomble *et al*. 2010). During these events, the kinetoplast has been duplicating, and it shortly after segregates into two resolvable structures, temporarily linked by a filamentous structure named the nabelschnur (Gluenz *et al*. 2011). Nuclear S-phase is completed sometime after that, and the duplicated DNA is segregated into two daughter nuclei in a closed mitosis (Ersfeld and Gull, 1997).

Throughout this time, the new flagellum has been extending towards the anterior end of the cell, using the old flagellum as a guide (Moreira-Leite *et al*. 2001; Briggs *et al*. 2004; Hughes *et al*. 2013). The newly-assembled axoneme and paraflagellar rod within the flagellum are paralleled by construction of the new flagellum attachment zone (Vaughan *et al*. 2008; Sunter *et al*. 2015; Zhou *et al*. 2015). A major difference between procyclic and bloodstream form *T. brucei* is the extent to which the new flagellum elongates along the old one – in procyclics, a ‘stop point’ is reached around 60% of the way along the old flagellum, with subsequent growth of the flagellum being driven by backwards extension (Davidge *et al*. 2006). In bloodstream form cells, the new flagellum continues tracking along the old one almost as far as the anterior tip of the cell body (Hughes *et al*. 2013). In either position, it is thought that the process of cytokinesis is triggered once the new flagellum attachment zone completes its assembly and contacts an anterior portion of the plasma membrane. Cytokinesis involves formation of a division fold along the anterior–posterior axis, and subsequent ingression of a cleavage furrow along this fold in an anterior-to-posterior direction to leave the two daughter cells joined by a cytoplasmic bridge at their posterior ends (Wheeler *et al.* 2013*b*). Unusually, in trypanosomes the actomyosin system is not involved in cytokinesis (Shi *et al.* 2000; García-Salcedo *et al*, 2004). As in other eukaryotes, however, the whole cell cycle process is orchestrated and coordinated by an interplay between several mitotic kinases, of which polo-like kinase (PLK) and aurora kinase are probably the best known (Hammarton, 2007; Li, 2012). The existence and placement of cell cycle checkpoints are not as well-known as in other model organisms, and it appears likely that trypanosomes have dispensed with some of those present in the opisthokont (animal and fungi) lineages (Hammarton *et al*. 2003). One of the few known examples of a specific cell cycle checkpoint in *T. brucei* monitors the synthesis of the predominating surface glycoprotein (Sheader *et al*. 2005). RNAi-mediated depletion of the surface glycoprotein triggers a precise arrest prior to cytokinesis.

Knowledge of the cell division cycle of *T. brucei* is not just of use for understanding of its basic biology. It is also required for determining the mode of action of existing or in-the-pipeline drugs, determining the mechanisms of drug resistance, and for the identification of possible new pathways for pharmacological targeting. However, cell division cycle analysis in *T. brucei* is currently a very labour-intensive process and could benefit from more standardization and automation. The ability to carry out automated cell division cycle analysis would be of obvious benefits not only to pure but also to applied research, allowing more refined analysis of small molecule screens and forward RNAi screens, amongst other applications. An additional complication for these screens and analyses is the fact that *T. brucei* populations grow asynchronously, and methods of synchronizing them remain somewhat time-consuming and inefficient. In the following sections, the existing methods for cell division cycle analysis and cell synchronization of *T. brucei* will be summarized. This will be followed by a consideration of candidate methods for global analysis of the trypanosome cell division cycle, and the contribution that automated, high-throughput analysis can make. Finally, a new tool to unify these approaches is proposed: *in silico* synchronization (ISS).

## CELL DIVISION CYCLE ANALYSIS IN *T. BRUCEI*

### The cell biology pipeline

**Fig. 3.**
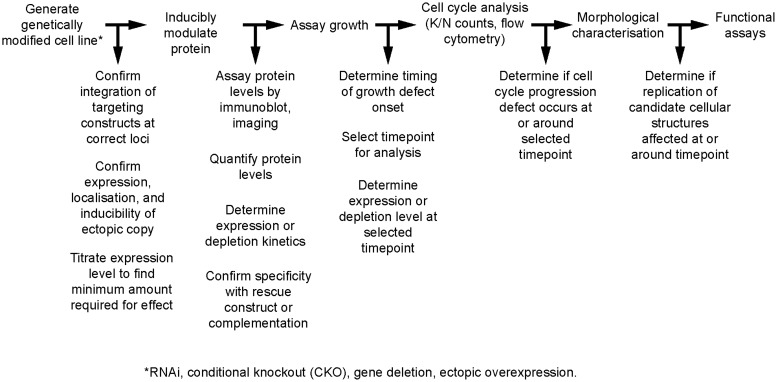
The cell biology pipeline. A generalized pipeline for the molecular cell biology analysis of a genetically modified cell line is shown. The pipeline is principally written for analysis of an RNAi or conditional knockout (CKO) cell line, but is applicable also to gene deletion and ectopic overexpression cell lines. Not every control is applicable for every type of cell line. Note that this assumes that the localization of the target protein has already been validated.

Cell division cycle analysis in *T. brucei* is typically carried out to characterize the effect of depletion of a protein of interest. Depletion is usually carried out using RNAi directed against the target protein, or through construction of a conditional knockout cell line in which a single ectopic allele is under regulated and inducible expression (Wirtz *et al*. 1999; LaCount *et al.* 2000; Wang *et al.* 2000; Alibu *et al*. 2005) (Fig. 3). After the generation of the required genetically modified cell line, it is vital that a number of control experiments are carried out and reported in order to verify its genotype and properties. These include confirming the integration of targeting constructs at the intended loci, confirming the expression, expression level and localization of any ectopic protein, and determining the kinetics of expression or depletion of the protein of interest (Fig. 3). Following validation of the cell line, the first step in its analysis is to assay for a growth defect upon modulation of the protein of interest, and select a timepoint or timepoints for further investigation. The choice of timepoint is made based on knowledge of the growth data and the level of protein depletion at that moment. Having identified the timepoint for analysis, the relative numbers of cells in different cell cycle states will be quantified using DNA labelling. Kinetoplast and nuclear DNA are labelled using DNA dyes such as DAPI or Hoechst, and the cells examined using either fluorescence microscopy or flow cytometry. Microscopic analysis of the cells allows the quantification of cells with one or two kinetoplasts (K) and one or two nuclei (N). The ordered progression of the cell division cycle, in which kinetoplast segregation precedes nuclear division, allows the easy partitioning of the cycle into three normal states: 1K1N, 2K1N and 2K2N. Accumulation of cells in one state or the appearance of cells with abnormal K/N numbers (for example, 1K0N, 0K1N and 1K2N) is immediately indicative of a possible cell division cycle effect. This approach has the advantage of decent resolution but is often done manually and is thus very labour intensive. Flow cytometry, while faster and more automated, permits only the sorting of G1/S and G2/M proportions of the cell cycle and the asynchrony of trypanosome populations further limits its resolution. Monitoring the actual duplication of DNA using for example BrdU is typically not considered unless replication of nuclear or kinetoplast material is the main focus of the project (Robinson and Gull, 1994).

A much greater problem, common to both approaches, is that the majority of the cell biological events associated with the cell division cycle – replication of the basal body, Golgi, ER exit site, flagellar pocket, flagellar pocket collar – are completed in the interval between the 1K1N and 2K1N states (Fig. 2). Around 70% of the cells in an asynchronous population of *T. brucei* will be 1K1N, yet this category covers everything from interphase cells, which have just completed cytokinesis, to cells with an almost completely replicated complement of organelles (Archer *et al*. 2011). Greater consideration of the cell biological states that lie between 1K1N and 2K1N cells is clearly needed.

Next, or coincident with the microscopic analysis of K/N states, is morphological characterization of the cells. At its most basic this involves imaging of live or fixed cells using transmitted light to determine whether there are any obvious abnormal phenotypic effects – partially or wholly detached flagella, enlarged flagellar pockets, impaired motility, and accumulation of cells showing arrested or impaired cytokinesis are a number of the most common examples. Further characterization can be accomplished using indirect immunofluorescence analysis with antibodies specific for various cellular structures or organelles. These assays allow determination of whether the duplication and/or segregation of major cellular features is affected; tabulation of these observations alongside the K/N counts can generally allow a rough estimation of where in the cell division cycle things begin to go awry. An important control is confirmation that the affected cells do indeed have lower levels of the target protein so that morphological changes can be directly correlated with impaired function.

It is vitally important that such assays are carried out as soon as phenotypic effects become manifest, ideally at or just before the point when the affected population begins to exhibit a growth defect. The seeming lack of some cell cycle checkpoints in *T. brucei* means that impaired cells almost never arrest at a particular stage – instead, organelle and DNA replication continues unabated in the absence of cell division, leading to the generation of polyploid ‘monster’ cells with multiple organelles and flagella. It is impossible to infer the original defect in such cells and the phenotypes are not informative. Only by determining what are the primary effects of a protein's loss can its likely function be inferred with any degree of confidence, and even then teasing apart direct and indirect effects can be challenging (Hammarton *et al.* 2007).

Once the morphological characterization is complete and an appropriate timepoint or timepoints for analysis have been determined, functional assays must be utilized (Fig. 3). These allow specific hypotheses concerning the protein's function to be tested. The wide availability of reverse genetics tools facilitating the deletion, overexpression, mutation, and truncation of genes in *T. brucei* is a distinct advantage of the system.

This morphological and functional characterization is extremely labour intensive and additionally carries a very large element of bias – only candidate structures/organelles are interrogated, so discovering unexpected phenotypic consequences of protein depletion (such as might be revealed in genetic suppressor screens) are not possible. The choice of timepoint is likewise subjective. An additional problem comes in the quantification of such assays. There exists no common standard for the conduct of these experiments, and the number of independent experiments (ideally at least three) is not always disclosed. Quantification is often carried out manually. Detailed understanding of statistics is not widespread in the life sciences, and the misuse of *P*-values to infer whether a result is real or not is as frequent here as elsewhere. For detailed practical recommendations on the proper processing and presentation of quantitative datasets, the reader is referred to the following articles: Cumming *et al*. 2007; Vaux, 2014; Klaus, 2016; Weissgerber *et al.* 2016. Undersampling (which compromises interpretation) and oversampling (which reduces efficiency) are also problems.

### Cell synchronization in T. brucei

The asynchronous growth of *T. brucei* populations has long been an impediment for accurate cell division cycle analysis. There appears to be small stochastic variation in the cell division cycle length in individual cells, resulting in a passive asynchronization of cell division cycle progression at a population level. Consequently, determining what are the primary effects of a protein's loss is made extremely difficult by the fact that such loss is initiated simultaneously in cells covering all stages of the cell cycle. Additionally, the depletion kinetics are slow and specific to individual proteins. In both RNAi and conditional knockouts, protein depletion depends on mRNA degradation or transcription cessation, followed by gradual protein loss as the target is turned over according to its intrinsic half-life. Mitotically-regulated proteins will exhibit very fast depletion as they are naturally degraded each cell cycle; most other proteins often exhibit very slow depletion as they are more stable, in general far more stable than their mRNAs. Thus, determining a protein's likely function currently requires consideration of how quickly it is simultaneously being lost from cells at each stage of the cell cycle – a task that is complex in the extreme.

To date, four main approaches have been utilized for synchronization of *T. brucei*, which will be considered in the following paragraphs. They are:

• starvation-induced,
• hydroxyurea-mediated,
• Vybrant DyeCycle,
• elutriation.

*Starvation-induced synchronization* is the simplest of the protocols. It requires growing cells to stationary phase, and then releasing them by dilution into fresh media (Gale *et al.* 1994). The absence of drugs or other manipulation is a plus, although allowing the cells to accumulate in stationary phase may carry its own set of consequences, as noted below. The cells do not achieve full synchrony, with a majority accumulating at G1, but a small number are still in G2/M phases (Gale *et al.* 1994; Archer *et al.* 2009, 2011). Upon release, the cells remain semi-synchronous for around 12 h, then resume asynchronous proliferation. Despite its simplicity, the principle drawbacks of this method are the incomplete synchronization achieved, the likelihood that the ‘synchronization’ is actually a recovery phase, and the high degree of variation between replicates (Archer *et al.* 2011). Starvation is also known to induce the formation of stress granules (Cassola *et al.* 2007; Fritz *et al.* 2015). The protocol has been almost exclusively applied to procyclic form cells. Use of starvation in bloodstream form cells has not been intensively pursued, although there are indications that some cell arrest occurs (Morgan *et al*. 1993). However, growing pleomorphic cell lines to stationary phase will induce the formation of stumpy cells, and in monomorphic cell lines a pseudo-stumpy morphology is produced. For the reasons mentioned above, the use of starvation-induced synchronization cannot be recommended.

*Hydroxyurea-mediated synchronization* has probably been the most popular of the techniques to date. Hydroxyurea inhibits ribonucleotide reductase, one of the enzymes involved in the synthesis of dNTPs (Hofer *et al*. 1997). The drop in cellular dNTP levels inhibits DNA replication, causing cells initiating or undergoing S-phase to accumulate. Early work using hydroxyurea on procyclic and bloodstream from *T. brucei* was discouraging however, with population growth being inhibited but DNA synthesis apparently not (Brun, 1980; Mutomba and Wang, 1996). This approach was revisited by the Englund laboratory in 2008, and a protocol for successful synchronization was reported (Chowdhury *et al.* 2008). The protocol was rapidly adapted for bloodstream form cells, with similar results (Forsythe *et al.* 2009). The exact mechanism remains unclear, as kinetoplast S-phase is completed while nuclear division is strongly inhibited, leading to an accumulation of 2K1N cells. Synchronization is thus achieved by slowing progression through the cell cycle, rather than arresting it. Once released from the block by hydroxyurea washout, the cells synchronously progress through the remainder of the cell cycle, before gradually becoming asynchronous over the course of the subsequent cycle. The hydroxyurea protocol is simple and appears to be nontoxic; however, its principle drawback is the relatively late stage at which cell cycle slowdown occurs – 2K1N. Most organelle and cellular structure replication has already occurred at this stage, which greatly limits the utility of the protocol for studying earlier events in the cell division cycle (Fig. 2). The relatively short time period in which the cells remain synchronized limits the opportunity for analysis. The use of chemical treatments generally to synchronize cells has been discouraged (Shedden and Cooper, 2002). The principle objection to chemical (or starvation)-induced synchronization is that it is often not clear that true synchronization has been achieved. Furthermore, the treatment may itself alter gene expression patterns.

*Vybrant DyeCycle* protocols are not true synchronization methods but instead produce selective enrichment of different cell cycle states. They involve the use of commercial DNA dyes (Thermo Fisher Scientific) coupled with fluorescence-activated cell sorting of the labelled cells. Vybrant DyeCycle Orange has been successfully used in procyclic cells (Siegel *et al*. 2008*a*, *b*). Vybrant DyeCycle Violet was subsequently used in bloodstream form cells (Kabani *et al*. 2010). Despite their simplicity and applicability to both procyclic and bloodstream form cells, the protocols are yet to be widely-adopted by the trypanosome research community. One key drawback is low yield, which reduces the number of cells available for downstream analysis. Arguably more important though is the low resolution afforded by fluorescence-activated cell sorting of *T. brucei*. In *T. brucei*, nuclear S-phase initiates before segregation of the duplicated kinetoplast DNA (Woodward and Gull, 1990; Siegel *et al.* 2008*b*; Kabani *et al*. 2010). Thus, selection of G1 cells will yield a mixed population of ‘true’ 1K1N cells in which kinetoplast duplication has not yet begun, and late 1K1N cells in which kinetoplast duplication is almost complete. As noted earlier, replication of many cytoplasmic organelles will already have occurred within this window (Fig. 2). Similarly, selection of G2/M cells yields a mixed population of late 1K1N cells, 2K1N cells and 2K2N cells. However, probably the principle objection is that the effect on the cells after labelling with the dye and sorting is unclear, with bloodstream form cells showing a significant delay in growth rates after the procedure (Kabani *et al.* 2010). The effect of the dye on cell division cycle progression in procyclic cells post-sorting has not been reported.

*Elutriation* is a mechanical process in which cells are sorted on the basis of size and density using a counterflow mechanism. Centrifugal forces are used to sediment cells towards the outer edge of an elutriation chamber, while an inward flow of buffer solution produces a counterflow drag force that selectively sorts cells within the population towards the centre of the chamber. By modulating the centrifugal force and buffer flow, different sizes of particles can be eluted from the chamber according to their individual sedimentation properties. A ‘double-cut’ elutriation (DCE) protocol was recently developed in a landmark paper from the Clayton laboratory (Archer *et al.* 2011). This protocol involves first sorting an asynchronous population to obtain the largest set of cells – i.e. cells at a relatively late stage of the cell division cycle and soon to undergo cytokinesis. These cells are then cultured for around 1 h, and subsequently subjected to a second round of elutriation. The brief incubation period allows time for the cells to undergo cytokinesis, and the second round of elutriation allows selection of the smallest cells, namely those in early G1 phase. The cells remain synchronized for around one cell cycle, and progress through the replication process at a normal rate. A crucial advantage of this protocol is that it allows analysis of the earliest events in the cell division cycle, unlike the hydroxyurea protocol. It also requires no chemical treatment, and although the approximate yield from the original population input is only 5% it can be scaled up easily. To date, the protocol has been applied in two elegant studies of the mitotic kinase TbPLK (Lozano-Núñez *et al.* 2013; McAllaster *et al.* 2015). In the first, the DCE protocol was combined with the use of an analogue-sensitive TbPLK to provide rapid and specific inhibition of kinase activity at different stages of the cell cycle, and consequent dissection of the requirements for TbPLK at those time points. The follow-up work combined the DCE and analogue-sensitive TbPLK approach with proteomics in order to identify putative binding partners and substrates of the enzyme. The DCE protocol clearly represents the best of the available protocols for cell synchronization in *T. brucei*, and the only one that is really compatible with biochemical and proteomic analysis. However, its main drawback is that to date it has been used solely on procyclic cells and it remains unclear whether it can be used for bloodstream form ones (Archer *et al*. 2011). The smaller size of bloodstream form cells relative to procyclic ones may impair their effective sorting in the procedure.

In summary, of the available protocols for cell synchronization in *T. brucei* described to date, none represents a truly optimal solution. Only DCE is able to produce synchronized populations of cells at early G1 phase, which progress through the cell cycle without an abnormal delay, and at present this technique has been optimized for procyclic cells only. None of the protocols is able to yield a population of cells that remain synchronized for longer than one cell division cycle. This latter fact already makes synchronization somewhat refractory to RNAi or conditional knockout approaches, as the depletion kinetics are too slow. Moreover, the rapid desynchronization of the artificially-synchronized cells is consistent with an intrinsic variation in the timing of cell cycle initiation and/or progression between individual cells in a clonal population. This stochasticity in the timings must have a biological, and possibly also an adaptive, reason. This begs the question as to whether artificial synchronization should be performed at all, despite its utility.

## APPROACHES FOR GLOBAL CELL ANALYSIS IN *T. BRUCEI*

Independent of whether cell synchronization is used or not, there remains a pressing need for a more global approach to cell division cycle analysis in *T. brucei*. As noted earlier, the focus on individual proteins that is typical of current cell biology approaches carries an inherent element of bias that may blind observers to unexpected effects. Two types of technique offer a degree of redress to this problem.

### Electron microscopy-based techniques (tomography, serial block face)

Two electron microscopy-based techniques – electron tomography and serial block-face scanning electron microscopy (SBF-SEM) – both present excellent solutions to the global analysis problem (Gluenz *et al*. 2015). Both techniques carry the enormous advantages of very high resolution and unbiased acquisition of data from all contrast-stained cellular structures and organelles simultaneously. Dual-axis electron tomography utilizes thick sections that are tilted in 1°–2° increments through a total path of around 120°–140°. Rotating the section through 90° allows the subsequent collection of data along an orthogonal axis, and the datasets can then be combined and aligned computationally to generate a three-dimensional image of the sampled cellular volume. SBF-SEM involves mounting an entire embedded sample within a SEM equipped with a diamond knife (Denk and Horstmann, 2004). The face of the block is imaged, and then sliced off with the knife. Sequential iteration of this process results in data acquisition through a large sample volume composed of many (hundreds or thousands) ultrathin sections. The two techniques are compatible in that sample preparation is the same. Separately and together, they have already resulted in a number of seminal papers on trypanosome morphology and ultrastructure, with the Vaughan laboratory's discovery of a cell surface groove guiding flagellum biosynthesis in bloodstream form cells a particular highlight (Hughes *et al*. 2013).

The principle drawbacks of both methods are the extremely demanding technical requirements in terms of both hardware and training. The hardware is expensive and specialized, which necessitates a considerable degree of institutional investment; sample preparation times – which will impact the quality of the data – are also relatively long. The need for advanced training and a period for the acquisition of tacit knowledge and experience mean that both techniques are off-limits to non-specialists. Tomography has a fairly small sampling volume, and the generation of a large number of independent datasets is very time-consuming.

### Fluorescence microscopy approaches

Fluorescence microscopy approaches are almost a counterpoint to the electron microscopy-based ones. The principle advantages of fluorescence microscopy are its short preparation time, and lower (although still not insignificant) technical costs. A key advantage relative to electron microscopy is the ability to observe live cells – chemical fixation can introduce artefacts, and the dehydration and plastic embedding steps required for many electron microscopy protocols causes cells to shrink. This makes the analysis of fixed cells (unavoidable for electron microscopy) ultimately less accurate for some morphological parameters, particularly cell length. Recent advances in super-resolution imaging offer the opportunity to close the gap on electron microscopy in terms of resolution, although these techniques are not for casual users. The main disadvantages of fluorescence microscopy approaches stem from its lower resolution compared with electron microscopy, and its requirement for the use of labels (either dyes, protein tags or antibody conjugates), which result in the selective illumination of one or only a small number of cellular structures. This unavoidably introduces an element of bias to the experiment design. In addition, its easy availability has resulted in a proliferation of protocols with consequently little standardization across the field and published data that are not always in agreement. A recent example are the two reported localizations for the phospholipid phosphatidylinositol-(4,5)-bisphosphate (Demmel *et al*. 2014; Cestari and Stuart, 2015). One paper reported a predominant localization at the flagellar pocket membrane; the other a diffuse localization throughout the plasma membrane and flagellar membrane. Publication of experiment metadata is not standard, and there are few enforced community standards for crucial procedures such as colocalization.

Both fluorescence and electron microscopy-based techniques allow a focus on cellular organelles and structures rather than individual proteins. While delineating the structure and function of individual proteins remains the ultimate goal of the reductionist approach to cell biology, proteins are generally not visible unless present in large complexes or macromolecular arrays. The higher visibility of organelles makes them a more appropriate focus, particularly in the context of analysing cell division cycle progression and phenotypes associated with it. It is worth noting too that correlated light and electron microscopy (CLEM) approaches potentially offer the best of both worlds.

In terms of scientific progression, an additional burden is the fact that work is currently carried out in multiple labs spread across disparate geographical locations. This results in an overall low efficiency in terms of rate of progress, and would benefit from ongoing studies being better able to bootstrap onto the conclusions of prior ones – more community-level organization, in other words. In this respect, the relatively small size and high interactivity of the *T. brucei* research network represents a distinct though relatively untapped advantage. Recent and ongoing community-level projects at different scales such as TriTrypDB and the TrypTag initiative offer encouraging signs that this nascent potential is beginning to be realized (Aslett *et al*. 2010; Dean *et al*., 2016; TrypTag, 2016).

## AUTOMATED CELL CYCLE ANALYSIS

Automated cell cycle analysis of *T. brucei* was pioneered in 2012 by the Gull laboratory, using a DNA double-staining technique and colour deconvolution method (Wheeler *et al.* 2012). The two DNA stains – DAPI, and propidium iodide or SYBR green – bind by interacting with the minor groove or intercalating between the base pairs, respectively. The method takes advantage of the different AT content of the kinetoplast and nuclear DNA, and the higher affinity of minor groove-binding dyes such as DAPI for AT-rich DNA. Consequently, the signals from the kinetoplast and the nuclear DNA can be distinguished based on their relative intensity. The high-throughput algorithm for automated image analysis developed using this labelling technique is capable of classifying asynchronous populations of *T. brucei* according to their cell cycle state (whether canonical or aberrant), and also provides information on cell length, width and shape based on phase contrast data. Importantly, the paper also provides a comparison between flow cytometry, manual counting and the algorithm in terms of accuracy. The methods all have a similar accuracy and precision, with flow cytometry the most precise and manual counting the least precise. However, flow cytometry is not capable of separately quantifying nucleus and kinetoplast DNA, which the other two approaches can do (Wheeler *et al.* 2012). Despite its obvious utility, there has been to date only one published instance of its application in *T. brucei* (Trindade and Rijo-Ferreira *et al*. 2016). The reasons for this are not clear, although the algorithm's requirement for high-quality phase contrast images may be a factor. It may also simply represent the time required for a new method to percolate into the standard toolbox of a research community. One possibility however is that the requirement for two DNA stains, when one is the *de facto* standard, introduces something of an activation energy barrier for adoption of the technique.

Besides the improvements in terms of reducing observer bias and increasing throughput, automated analysis carries another and potentially transformative benefit – the opportunity to derive cell division cycle progression from asynchronously-growing populations. A recent theoretical paper from Richard Wheeler on the ergodic principle makes this implication explicit (Wheeler, 2015). Ergodicity (from the Greek words ‘ergon’ meaning ‘work’, and ‘odos’ meaning ‘path’) was coined by Ludwig Boltzmann in the late 19th century as part of his work on statistical mechanics. In general terms, it describes dynamic systems in which the ensemble behaviour of the components of that system at one point in time (i.e. a snapshot) is similar to the behaviour of a single component of the system over a timecourse. This matches the behaviour of growing microbial populations – asynchronous cell cultures are in a quasi-steady state, with the proportions of cells in a particular category (for example, 1K1N, 2K1N and 2K2N) remaining approximately constant over time. This is due to the fact that the number of cells in each category will be directly related to the time spent in that state. However this also requires accounting for the effect of binary fission – the production of two daughter cells entails the ‘loss’ of one mother cell, so there are always twice as many cells entering the next cell cycle as there are ones undergoing the previous one. This results in an overrepresentation of early cell cycle events in the population, but can be easily adjusted for mathematically. Furthermore, cells progress from one category to the next in an ordered sequence (1K1N progresses to 2K1N, which progresses to 2K2N). As such, asynchronous populations contain information on all stages of the cell division cycle simultaneously, and static images of these populations are more information-rich than single images of synchronized cells. For a more detailed explanation of the weak and strong ergodic assumptions that such analyses require, the reader is directed to the article mentioned earlier (Wheeler, 2015). The mathematics underpinning the disentanglement of such datasets has been established for more than forty years, but has only recently begun to be applied to quantitative cell biology (Kafri *et al*. 2013). The continuing advances in computing power that have become available to the scientific community over the last decade or so now make it possible to envision a new approach in *T. brucei* cell division cycle analysis, which we here refer to as ‘ISS’.

## *IN SILICO* SYNCHRONIZATION

ISS would represent a fast, simple, and cost-effective solution to cell division cycle analysis that fulfils all of the requirements articulated above. By using multiple continuous variables (initially DNA content and flagellum length) and ergodic analysis, it will be possible to provide a precise timeline for cell cycle progression such as has already been achieved in *Leishmania* (Wheeler *et al*. 2011). This timeline can be derived from asynchronously-replicating populations and thus permits a fast and ISS without the need for chemical treatments or labour-intensive handling. The use of computational methods also means that the populations can be observed for as long as wished, without the short time windows engendered in current synchronization protocols by the rapid loss of synchrony.

Importantly, once this timeline has been established, it will enable the addition of further parameters with increasing ease. Morphological details such as cell length, width, surface area and volume (obtained using fluorescence labelling of the cell plasma membrane) can be integrated by bootstrapping observations of their variability in asynchronous populations alongside one of the prior variables (DNA content, flagellum length). Automated analysis will increase throughput and standardize quantitation of the data. Fluorescence labelling of different organelles (using immunofluorescence, fluorescent protein-tagged reporters and fluorophore-conjugated cargo molecules) can then add successive layers of detail in order to gradually provide an unbiased overview of the replication of all cellular organelles and structures. The drawbacks in fluorescence labelling noted in the section above can be compensated for by the ease and speed of data acquisition. ISS would by no means be incompatible with the use of SBF-SEM or even electron tomography – these techniques could improve the resolution still further.

The progressive accumulation of time-indexed replication of cellular organelles and structures should eventually make the generation of a full mathematical model for the *T. brucei* cell division cycle feasible. This standardized *in silico* cell would permit interrogation of a number of issues that are currently challenging. These would include but not be restricted to: the interplay between organelles during their replication; the relative timing of events during the cell cycle; finer temporal resolution of rapid events; delineation of membrane-membrane contacts between organelles.

Intuitively, the model would be compatible with analysis of both RNAi phenotypes and drug treatments. ISS of RNAi-induced or drug-treated populations using only DNA labelling would pinpoint with high temporal resolution the exact stage of the cell division cycle at which a phenotype begins to become manifest, though it should be noted that accounting for the depletion kinetics inherent to RNAi will be computationally challenging. Reference to the *in silico* cell would enable identification of the cellular organelles undergoing replication at that time point and other putatively-affected cellular processes. This should make the analysis of pleiotropic phenotypes far more feasible than is currently the case. Standard reverse genetics analysis of the RNAi phenotypes associated with individual proteins will be enriched by knowledge of the cell cycle behaviour of the structures and organelles they are associated with.

Ultimately, it is possible to envisage the *in silico* cell generated from accumulated ISS data becoming an online portal for community-level use and annotation. This would allow the rapid integration of additional datasets and ongoing refinement, with no alteration to existing lab protocols required. As noted previously, the relatively small size of the *T. brucei* research community and its high level of interactivity and communication make this an achievable goal that would be more difficult for less social fields.

### Concluding remarks

In this review, the existing methods for cell division cycle analysis and synchronization in *T. brucei* have been summarized and evaluated. Techniques for global cell analysis have been compared, and the promise offered by automated and high-throughput computational methods highlighted. The adoption of ISS as a new tool for cell division cycle analysis has been advocated. The advent of single-cell transcriptomics (and potentially even proteomics) is a parallel development that will also enable the analysis of asynchronous populations. By taking advantage of ongoing advances in computational biology, this perhaps offers an unprecedented opportunity for hearing trypanosome bodies talk.
